# Effective Computational Model for Determining the Geometry of the Transition Zone of End Coils of Machined Springs, Enabling Efficient Use of the Spring Material

**DOI:** 10.3390/ma17071540

**Published:** 2024-03-28

**Authors:** Krzysztof Michalczyk, Rafał Grzejda, Andrzej Urbaś, Patryk Różyło

**Affiliations:** 1 Faculty of Mechanical Engineering and Robotics, AGH University of Krakow, al. Mickiewicza 30, 30-059 Krakow, Poland; 2 Faculty of Mechanical Engineering and Mechatronics, West Pomeranian University of Technology in Szczecin, al. Piastow 19, 70-310 Szczecin, Poland; rafal.grzejda@zut.edu.pl; 3 Faculty of Mechanical Engineering and Computer Science, University of Bielsko-Biala, 2 Willowa Str., 43-309 Bielsko-Biala, Poland; aurbas@ubb.edu.pl; 4 Mechanical Engineering Faculty, Lublin University of Technology, 36 Nadbystrzycka Str., 20-618 Lublin, Poland; p.rozylo@pollub.pl

**Keywords:** helical spring, machined spring, closed end coils, stress concentration factor, spring design, finite element method

## Abstract

This paper presents an analysis of the effect of the geometry of the end-coil transition zone on the material stress state of a machined compression spring with a rectangular wire cross-section. The literature relationships for determining the stresses in rectangular wire compression springs neglect the effects associated with the geometry of this zone. A series of non-linear numerical analyses were carried out for models of machined compression springs with a wide range of variation in geometrical parameters. The results of these analyses were used to develop a computational model to estimate the minimum value of the rounding radius *ρ_min_*, which ensures that the stresses in this zone are reduced to the level of the maximum coil stresses. The model is simple to apply, and allows the radius *ρ_min_* to be estimated for springs with a spring index between 2.5 and 10, a helix angle between 1° and 15°, and a proportion of the sides of the wire section between 0.4 and 5.

## 1. Introduction

Coil springs are the most commonly used spring link in mechanical engineering. They are an essential element in the suspension of rail vehicles, motor vehicles, support systems for vibrating machinery, and are found in position-change mechanisms, return-locking mechanisms, valves, mechanical seals, and many other applications [1,2,3,4]. In systems with high-cycle alternating loads, compression springs are used, while tension springs are avoided. This is due to the shot-blasting capability of compression springs. This treatment significantly increases the fatigue strength of the spring wire. For instance, based on EN 13906-1 [5], the torsional fatigue strength of a patented spring wire, 10 mm in diameter, made in accordance with EN 10270-1 [6] after shot-blasting is approximately 410 MPa, whereas without shot-blasting treatment, this strength is only 320 MPa. Hora and Leidenroth [7] and Berger and Kaiser [8] even state that the surface condition of the spring wire has a much greater influence on its fatigue strength than its material properties. Tension springs cannot be effectively shot-blasted due to the adhesion of adjacent coils in the unstressed state, while if the wire breaks, the system completely loses integrity. In addition, as shown in the paper [9], the maximum stress in hooks of such springs can achieve high values. For these reasons, even in systems with tensile forces, compression springs with appropriate intermediate elements are used for responsible devices.

The most common industrially used compression springs are made of wire with a round cross-section [10,11], which provides the most efficient use of material due to the tangential stress distribution caused by wire torsion. However, there are a number of applications where springs with a rectangular wire cross-section are preferable. Springs with a rectangular wire cross-section have a high energy storage capacity, high stiffness, and a small size, so they are applicable in the automotive industry [12], in automotive vehicle suspensions [13], and are also commonly used in stamping machines [14]. These springs are also applied as prone couplings and as flexible connectors in manipulators, including surgical robots [15]. The favourable characteristics of rectangular wire springs have led to attempts to make them using 3D printing technology [16] and to make them from composites [17,18].

Springs with a rectangular wire cross-section can be made by coiling from wire or by cavity machining. Rectangular wire is most commonly used to coil these springs. When coiling a helix from a wire with a rectangular cross-section, the wire usually adopts a near-trapezoidal shape, which reduces the space efficiency and energy storage capacity of the spring [19]. Tsubouchi et al. [20] proposed a new technology involving the coiling of a round wire spring, which is then subjected to presetting. During the presetting process, adjacent coils of the spring are interlocked. This process is then continued, resulting in plastic deformation of the wire caused by high pressures between the pressed coils, the cross-section of which takes the shape of a rectangle with two rounded sides. The authors of the cited paper indicate that this treatment increases the fatigue strength of the spring. However, this leads to a spring with less axial stiffness due to a reduction in the original axial moment of inertia of the wire cross-section.

**Figure 1:**
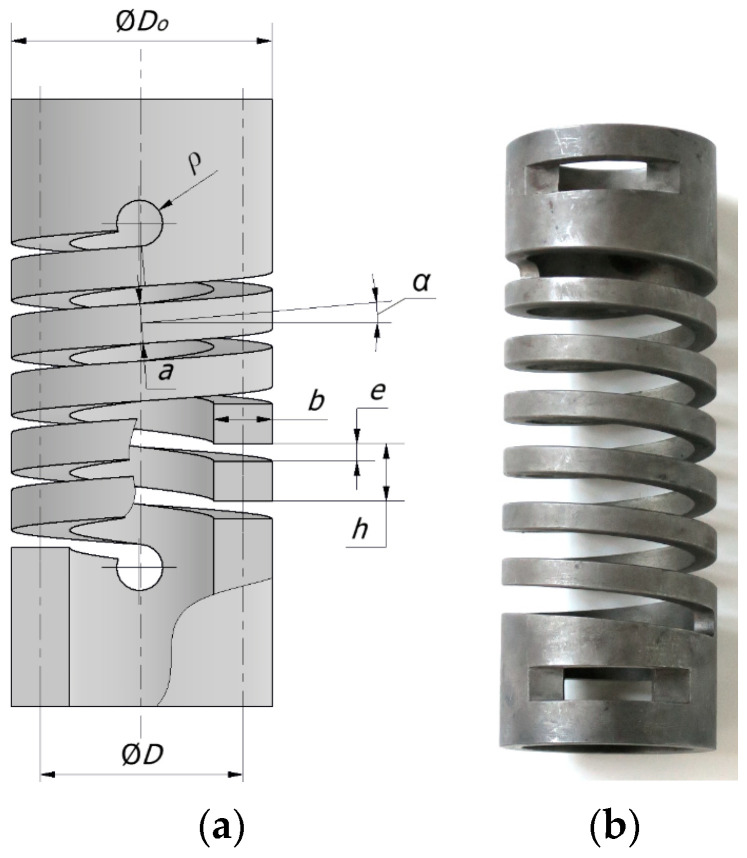
Closed-end coil spring machined from a cylinder: (**a**) sketch with main geometrical parameters; (**b**) photo of the spring.

3D printing, electro-discharge machining, laser machining, or milling technologies make it possible to produce springs with a much wider range of geometrical parameters than spring coil technology. In addition, these springs can be made from a much wider spectrum of materials, including spring-brittle materials, than with coiling technology. A significant advantage of machining is the possibility to produce springs with a very small spring index *C*, i.e., the quotient of the nominal spring diameter *D* to the wire thickness measured in the radial direction *b*. A relatively new design of machined spring is a closed-end coil spring made from a cylinder. A drawing of such a spring with the main geometrical parameters is shown in Figure 1a. Figure 1b illustrates an example of such a spring. As can be seen, the end parts of the spring can be shaped to facilitate assembly, which significantly increases the application possibilities. The spring can thus be used in compression, tension, bending, and torsion.

In the literature, relationships can be found to calculate the maximum stresses developed in the spring material under axial loading [13,21]. However, they do not take into account effects related to the way the coils of machined springs are terminated, such as those shown in Figure 1. Moreover, these relations are derived on the basis of simplifying assumptions, and no comprehensive analysis of their accuracy for a wide range of parameter variations is presented anywhere, even for classical coil springs made of rectangular wire. An important geometrical parameter for springs of this type is the radius *ρ* of the rounded end of the coil, shown in Figure 1a. If it is too small, it will cause an increase in stress in the spring material during operation and, as a result, reduce the static and fatigue strength of such a spring. The value of this radius also affects the stiffness of the spring, especially for springs with a small number of coils. Using a rounding radius larger than necessary for stress reduction results in a spring with increased dimensions and reduced stiffness. The reason for this is that, in order to provide stable support for the spring, its retaining surfaces should be in the form of solid rings. This compromises the spring’s performance properties due to, among other things, increased weight and required installation space. The lack of a model to determine the correct value of this radius makes it difficult to correctly select parameters when selecting or designing such a spring.

The aim of this study was to provide a comprehensive analysis of the stresses in the material of a machined coil spring with closed-coil ends. This analysis was carried out using numerical methods for a wide range of variations in the geometrical parameters of the springs. The first objective of this analysis was to determine the minimum value of the rounding radius *ρ_min_*, ensuring that there is no stress concentration at the ends of the coils. The second objective of the analysis was to develop an easy-to-use computational model to estimate the value of the rounding radius *ρ_min_* for machined springs with arbitrary geometric parameters within the assumed range of variation. This analysis and the developed computational model will allow the springs to be designed with efficient use of material and the application of relationships known from the literature for their calculation, which neglect the effects associated with the geometry of the transition zone of the end coils.

## 2. Materials and Methods

### 2.1. Parameter Variation Range of the Analysed Spring Models

In order to obtain results that are representative of a wide spectrum of springs, the following ranges of parameter variation were assumed:

- Proportion of the sides of the wire section (the so-called aspect ratio) $\bar{b}=\frac{b}{a}$, $\bar{b}\in \left\{5/1,\;2.5/1,\;1/1,\;1/2.5\right\}$.
- Spring index $C=\frac{D}{b}$, $C\in \left\{2.5,\;5,\;10\right\}$.
- Helix angle $\alpha ={\mathrm{tg}}^{-1}\left(\frac{h}{\pi D}\right)$, $\alpha \in \left\{1{}^{\circ},5{}^{\circ},\;10{}^{\circ},\;15{}^{\circ}\right\}$.
- Number of coils n, $n\in \left\{1.5,\;2.5,\;3.5,4.5\right\}$.

**Figure 2:**
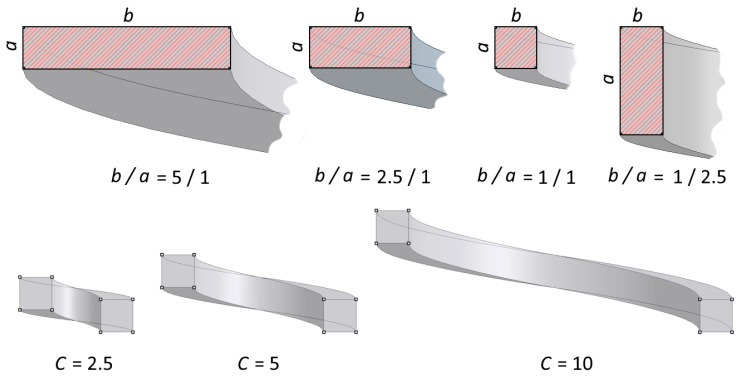
Graphical interpretation of the values of the aspect ratio $\bar{b}$ and the spring rate C.

Figure 2 provides an illustration of the values of the analysed parameters *C* and $\bar{b}$.

The range of variation of the parameter *ρ* was not possible to determine in the first stage of the analysis. It was assumed that a minimum value of the relative radius $\bar{\rho }=\rho /a$ would be sought, ensuring that the stresses in the end region of the coils would be no greater than the stresses in the prismatic part of the coils. Such a value for the relative radius is denoted by ${\bar{\rho }}_{min}$ in the remainder of the paper. The search was carried out using an iterative method, with a step of $\Delta \bar{\rho }=0.1$.

### 2.2. Development of a Parametric Numerical Model

**Figure 3:**
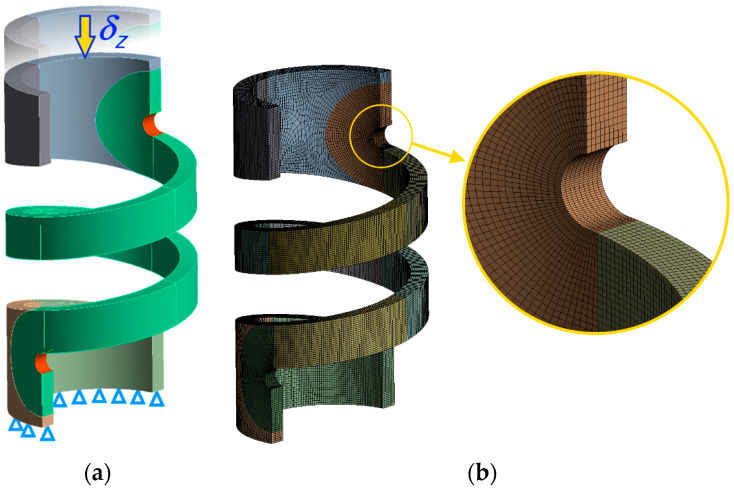
Example spring model: (**a**) solid model with selected parameters and boundary conditions; (**b**) discretisation of the model by the finite element method with enlargement of the rounded end of the coil.

In all analyses carried out, models with parameterised values for the size of the finite element mesh (FE mesh) were used. Solid models of the springs were developed in the DesignModeler module of the ANSYS 2022 R1software [22] and their dimensions were parameterised. They were subdivided into smaller solids, allowing a high-quality FE mesh to be obtained. In the spring models, the areas of active coils and groove ends were extracted. Figure 3a shows an example of a solid model of a spring with selected geometrical parameters, together with the adopted boundary conditions. Figure 3b, on the other hand, shows the corresponding model created in the finite element method (FEM) convention [23].

In Figure 3a, the green colour indicates the active coil areas. In these areas, models have been discretised using quadratic 20-node hexahedral elements. Discretisation using these elements requires more work and is more difficult to automate than using tetrahedral elements, but this type of finite element generally gives more accurate results for stresses and strains than quadratic elements [24,25]. The hexahedral elements are also more reliable in terms of the skewness parameter than the tetrahedral elements [26]. The value of the skewness parameter was tested for each spring model, and in all cases its average value across the model did not exceed 0.25. The size of the finite elements in the volume marked in green in Figure 3a was dependent on the smaller of the wire cross-sectional dimensions, the length of which, as mentioned above, was 1 mm in all analyses. For springs with an aspect ratio of $\bar{b}=5/1$, the finite element size was set to 1/6 of the shorter of the sides, while for springs with aspect ratios of $\bar{b}=2.5/1$, $\bar{b}=1/1$ and $\bar{b}=1/2.5$, the finite element size was set to 1/9 or 1/12 of the shorter of the sides to ensure a high quality of the FE mesh in each case. The dimensions *b* and *a* were normalised, assuming in all analyses that the shorter side has a value of 1 mm. The red colour indicates the areas of groove rounding, where the set element size ensured that the rounding arc was divided into 18 elements in all the analyses carried out. The total number of mesh nodes in the spring models ranged from about 0.3 × 10^6^ for the spring models with the smallest index and the smallest number of coils to about 3 × 10^6^ for the spring models with the largest index and the largest number of coils.

The spring models described above were subjected to axial compression analyses. Boundary conditions were assumed in accordance with Figure 3a. The lower resisting surface of the spring was restrained, while the upper surface was deprived of all degrees of freedom, except translation along the spring axis. In this direction, the axial displacement $\delta_{Z}$ of this surface was given a value equal to 0.25 of the total inter-coil clearance:

(1) $$\delta_{Z}=-0.25en$$

where *e* is the clearance between two coils measured in axial direction, as shown in Figure 1a, and *n* is the number of coils.

In the analyses, large-scale deformations were considered, and material properties representative of steel were assumed: Young’s modulus *E* = 200 GPa and Poisson’s ratio *ν* = 0.3. The maximum equivalent von Mises stresses were measured and the location of their occurrence was determined, and the reaction force *P_Z_* was read in the axial direction, on the lower retaining surface, corresponding to a given displacement of each spring by $\delta_{Z}$. In total, more than 350 large-scale deformations analyses were performed.

### 2.3. Numerical Verification of the Model Reliability in Terms of Stress Representation

As it was not possible to precisely measure the stresses in the rounding region experimentally, verification of the validity of the FEM-based model was carried out using results taken from the literature. As bending stresses in the spring wire in the rounding region represent a significant component of all stresses, the model of bending of a stepped flat bar with shoulder fillets, for which precise formulas for stress concentration factors (SCFs) can be found in the literature, was therefore used. Noda et al. [27] analysed stress concentration of round and flat stepped bars by the body force method (BFM) and proposed new formulas of SCFs calculation with better than 1% accuracy. These relationships give more accurate SCFs values than those found in the widespread literature such as [28,29], where SCFs values can vary significantly to the detriment of calculation safety. The present study compares the SCFs calculated in [27] with the values calculated from the FEM-based analyses carried out with the model settings and analyses described in Section 2.2. This comparison is for reference, as it was only made after all the axial compression analyses of the springs had been carried out to determine the value of ${\bar{\rho }}_{min}$ for each spring. The minimum value of the relative rounding radius when the finite element size was set to 1/6*a* was ${\bar{\rho }}_{min}=1$, and when the finite element size was set to 1/9*a* the value was ${\bar{\rho }}_{min}=0.2$ (with *a* being defined in Figure 1a).

**Figure 4:**
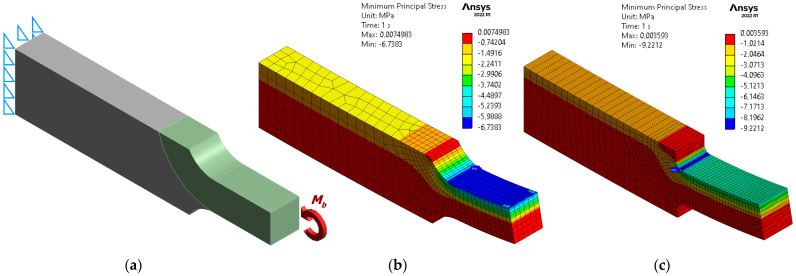
Calculation results for exemplary flat stepped bar models: (**a**) restraint and loading conditions; (**b**) contour plots of minimum principal stress for the model with $\bar{\rho }=1$; (**c**) contour plots of minimum principal stress for the model with $\bar{\rho }=0.2$.

Figure 4 presents the results of FEM-based bending analyses of stepped flat bars with rounded shoulders. The dimension values shown in Figure 4 are: *a* = 10 mm, *b* = 10 mm, *W* = 20 mm. The bending moment *M_b_* was 1000 N·mm. The extreme nominal stress value on the compression side of the beams was −6 MPa.

A comparison of the values of the stress concentration factors obtained from the numerical analyses (*SCF_FEM_*) with the values of these factors taken from [27] (denoted by *SCF_BFM_*) is shown in Table 1.

The high agreement between the values of the SCFs calculated from the FEM-based analyses at the given FE mesh parameters and the reference values of these factors indicates the high accuracy of the FEM-based models used with regard to stress determination. As can be seen in Table 1, the largest relative difference ${∆}_{SCF}$ between the values of these factors for the model with $\bar{\rho }=0.2$ did not exceed 2.6%, and for the model with $\bar{\rho }=1$, the difference was 2.2%.

## 3. Results

### 3.1. Determination of ${\bar{\rho }}_{min}$ as a Function of the Other Geometrical Parameters of the Springs

**Figure 5:**
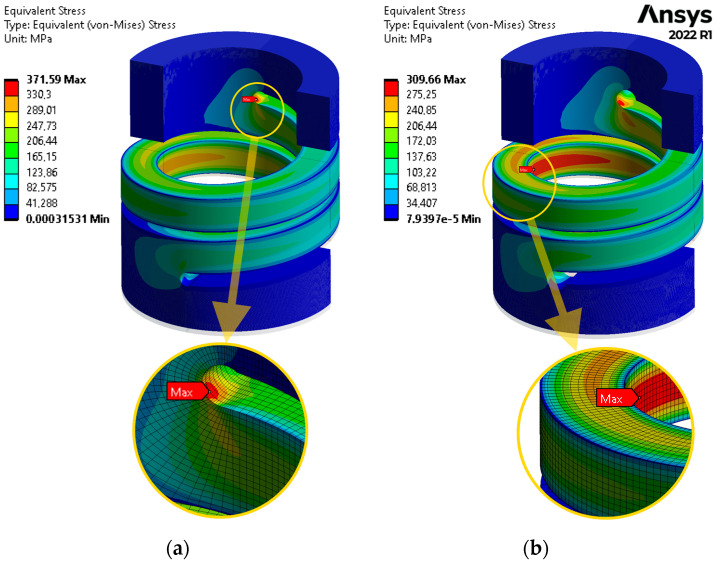
Contour plot of the equivalent stress for a spring model with the following parameters: *C* = 5, *α* = 5°, $\bar{b}=1/1$, *n* = 1.5 for: (**a**) $\bar{\rho }=0.2$; (**b**) $\bar{\rho }={\bar{\rho }}_{min}=0.3$.

Figure 5a,b shows an example results of an axial compression analysis of a spring model with the following parameters: *C* = 5, *α* = 5°, $\bar{b}=1/1$, *n* = 1.5.

Figure 5a presents a contour plot of the equivalent stress for a spring with a rounding radius $\bar{\rho }=0.2$, and Figure 5b shows an analogous plot for a spring with a rounding radius $\bar{\rho }=0.3$. Below the spring models, magnified sections of the FE mesh where regions of maximum stress occur are shown.. As mentioned above, the relative minimum radius ${\bar{\rho }}_{min}$ was sought in all cases with a step equal to 0.1. As can be seen, in the latter case, the maximum stress at the rounding point is smaller than at the prismatic part of the coils. Thus, for a spring with parameters *C* = 5, *α* = 5°, $\bar{b}=1/1$, *n* = 1.5, the minimum rounding radius ${\bar{\rho }}_{min}=0.3$. In the same way, the value of ${\bar{\rho }}_{min}$ was determined for all spring models, with the parameters given in Section 2.1.

When the relative difference between the maximum equivalent stress in the model with $\bar{\rho }={\bar{\rho }}_{min}$ and the maximum equivalent stress in the model with $\bar{\rho }$ smaller by 0.1 was less than 1% then the smaller of these radii was taken as ${\bar{\rho }}_{min}$. The resulting values are shown in Table 2, Table 3, Table 4 and Table 5. In the presentation of the results, some of these are not included. They concern combinations of such geometrical parameters of the springs, at which the clearance between coils takes a negative value, i.e., springs that are impossible to perform.

**Figure 6:**
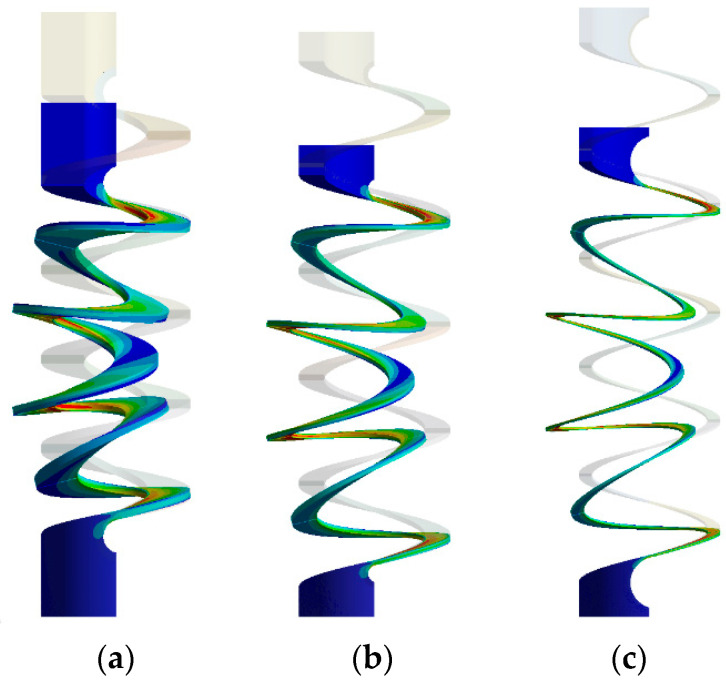
Examples of buckling of springs with the following parameters $\bar{b}=5/1$, *α* = 15° and *n* = 4.5 for: (**a**) *C* = 2.5; (**b**) *C* = 5; (**c**) *C* = 10.

For springs with certain parameters, buckling occurred at a given axial deflection equal to 0.25 of total clearance. Examples of such cases are shown in Figure 6. In such cases, buckling is indicated in the table instead of the ${\bar{\rho }}_{min}$ value.

Comparing the ${\bar{\rho }}_{min}$ values for springs with number of coils *n* = 2.5 (Table 3) with the corresponding ${\bar{\rho }}_{min}$ values for springs with *n* = 1.5 (Table 2), with the same values of the parameters $\bar{b}$, *C*, and *α*, it can be seen that there are only eight repeated ${\bar{\rho }}_{min}$ values and 26 cases where a change in the number of coils from 1.5 to 2.5 caused a change in the ${\bar{\rho }}_{min}$ value. Doing the same comparison for springs with *n* = 3.5 and *n* = 2.5, it can be seen that there are already 24 repeated values of ${\bar{\rho }}_{min}$ and only seven cases where changing the number of coils from *n* = 2.5 to *n* = 3.5 caused a change in the value of ${\bar{\rho }}_{min}$. Again, comparing the results for a spring with *n* = 4.5 and *n* = 3.5, it can be seen that there are already 27 repeated values of ${\bar{\rho }}_{min}$ and there are only four cases of variation in ${\bar{\rho }}_{min}$ with a change in the number of coils. Comparing the results in Table 4 and Table 5, it can be noted that a change in the value of ${\bar{\rho }}_{min}$ occurred for springs with the following parameters:

(a) *C* = 2.5, $\bar{b}=5$, *α* = 5°
(b) *C* = 5, $\bar{b}=5$, *α* = 5°
(c) *C* = 10, $\bar{b}=5$, *α* = 5°
(d) *C* = 10, $\bar{b}=2.5$, *α* = 15°

Additional FEM-based analyses were carried out for the above four cases, increasing the number of coils to *n* = 5.5. The analyses were performed with ${\bar{\rho }}_{min}$ values as determined for *n* = 4.5. These analyses showed that the ${\bar{\rho }}_{min}$ values in cases (a) and (b) did not change after increasing the number of coils to *n* = 5.5. In case (c), at the same value of ${\bar{\rho }}_{min}$ as for the spring with *n* = 4.5, the maximum stress in the transition zone exceeded the maximum stress in the prismatic part of the coils by 1.2%. In case (d), buckling of the spring occurred.

### 3.2. Development of a New Computational Model Approximating the Relationship between ${\bar{\rho }}_{min}$ and Other Geometrical Parameters of the Springs

In order to establish an easy-to-apply relationship to estimate the value of ${\bar{\rho }}_{min}$ for any other geometrical parameters of the springs, the computational model developed is based on certain simplifications. Simplifying assumptions were made in order to obtain a stable computational model giving results with high accuracy, both for intermediate parameter values between those investigated using FEM-based analyses, as well as for geometric parameter values outside the investigated range. In the first step, the values of the parameter ${\bar{\rho }}_{min}$ obtained from FEM-based analyses were analysed. As recognized in Section 3.1, these analyses showed that, for fixed values of *C*, $\bar{b}$, and α, increasing the number of coils *n* is accompanied by a decreasing change in the value of ${\bar{\rho }}_{min}$.

By analysing the results in Table 2, Table 3, Table 4 and Table 5, it can be seen that the lowest determined value of ${\bar{\rho }}_{min}$ is 0.2. However, this value occurred only five times and only for springs with *n* = 1.5. In the other cases, the lowest value of ${\bar{\rho }}_{min}$ is 0.3. Therefore, the values of ${\bar{\rho }}_{min}=0.2$ were replaced by ${\bar{\rho }}_{min}=0.3$ in the approximation process. In addition, it can be seen that for springs with aspect ratios $\bar{b}=1/1$ and $\bar{b}=1/2.5$, changing the helix angle α in most cases does not change the value of ${\bar{\rho }}_{min}$. In such cases, the approximation process extrapolated the results to smaller values of angle α in order to increase the stability of the sought function.

**Figure 7:**
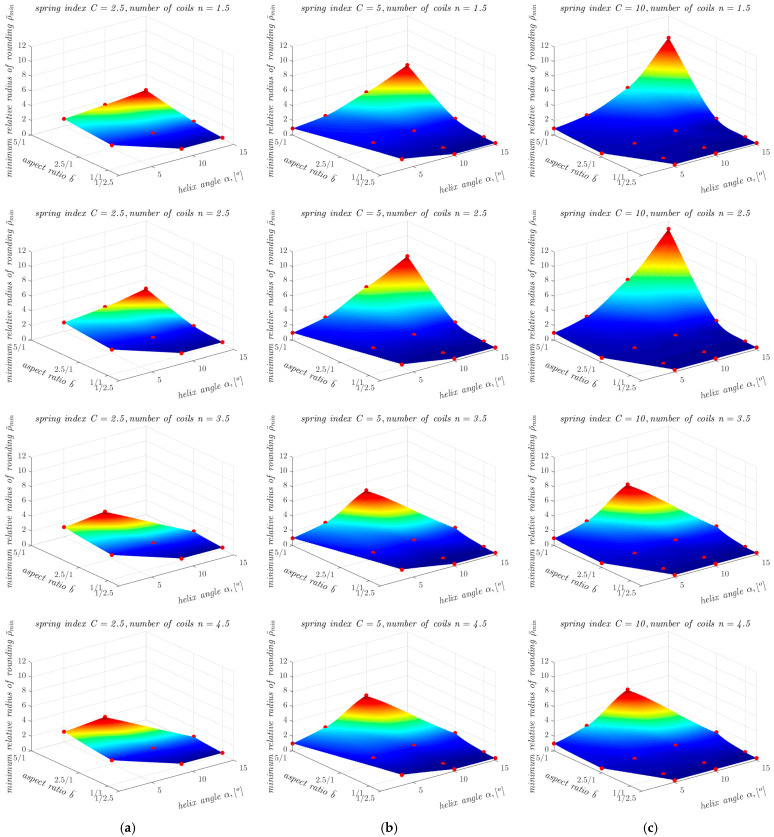
Dependence of ${\bar{\rho }}_{min}$ on the aspect ratio $\bar{b}$ and the helix angle *α* in the case of springs with the spring index *C* equal to: (**a**) 2.5; (**b**) 5; and (**c**) 10 (the red dots correspond to the points collected in Table 2, Table 3, Table 4 and Table 5, the absence of ${\bar{\rho }}_{min}$ values for springs with *n* = 3.5 and *n* = 4.5 for $\bar{b}=5$ and α = 15° is due to the occurrence of buckling).

The determination of ${\bar{\rho }}_{min}$ as a function of other geometrical parameters of the springs is also illustrated in the graphs shown in Figure 7. As can be seen from Figure 7, the dependence of ${\bar{\rho }}_{min}$ on aspect ratio $\bar{b}$ and on helix angle *α* is non-linear, so an attempt was made to approximate ${\bar{\rho }}_{min}$ for all 12 combinations of parameter values *α* and *n* using a non-linear function. The search for a suitable form of the approximating function was conducted using a trial-and-error approach. The approximations were carried out in the MATLAB environment (MathWorks, Natick, MA, USA) using the non-linear least squares method. Of all the closed-form functions tested (including exponential functions with different numbers of coefficients, polynomial functions with different numbers of coefficients and different powers of the variables, and products of functions of one variable), the following exponential model of general form showed the best agreement with the data for all 12 cases:

(2) $${\bar{\rho }}_{min}=c_{1}e^{\left(c_{2}\alpha +c_{3}\bar{b}\right)}+c_{4}$$

Additionally, an important advantage of this form of the function is the small number of its coefficients *c_i_* (for *i* = 1, 2, 3, 4), which simplifies the subsequent approximation process, which involved determining the dependence of the coefficients of Equation (2) on the other two variables, i.e., on the spring rate *C* and on the number of coils *n*. The use of higher-degree polynomials [30] in the first approximation step, which can be prone to overfitting, could lead to poor generalisation performance.

The approximations were carried out using weights, with values inversely proportional to the value of ${\bar{\rho }}_{min}$. This way of approximation allowed us to obtain a model with a better agreement with the data for small values of ${\bar{\rho }}_{min}$. During the approximation process, modifications were made to the default coefficient constraints in order to stabilise the values obtained. Table 6 shows the values of the coefficients *c_i_* together with the coefficients of determination *R*^2^ and mean squared error (MSE) [31].

The next step in the development of the computational model was to determine the approximation formulas for the dependence of the coefficients *c_i_* on the spring index *C* and the number of coils *n*. After a number of tests, finally, a polynomial of second degree with respect to the spring index *C*, and of third degree with respect to the number of coils *n*, were used as approximating functions:

(3) $$c_{i}=p_{00}+p_{10}C+p_{01}n+p_{20}C^{2}+p_{11}Cn+p_{02}n^{2}+p_{21}C^{2}n+p_{12}Cn^{2}+p_{03}n^{3}$$

**Figure 8:**
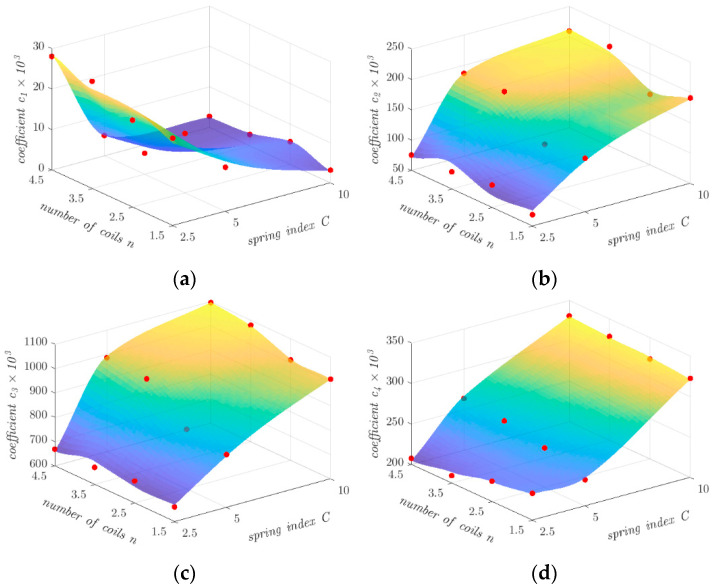
Dependence of the coefficients *c_i_* on the spring index *C* and the number of coils *n* for: (**a**) *c*_1_; (**b**) *c*_2_; (**c**) *c*_3_; and (**d**) *c*_4_.

Of all the functions tested, this form gives the best agreement with the data. Table 7 shows the values of the constants appearing in Equation (3), and Figure 8 graphically shows the values of the coefficients *c_i_* together with the functions approximating the dependence of the values of these coefficients on the spring index *C* and the number of coils *n*.

## 4. Discussion on the Accuracy of the Developed Computational Model

**Figure 9:**
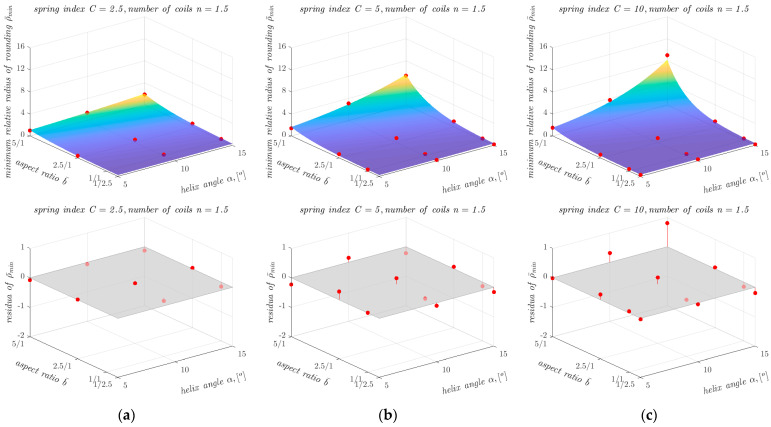
Dependence of ${\bar{\rho }}_{min}$ on the other parameters of the analysed springs (for *n* = 1.5), together with points showing the values of ${\bar{\rho }}_{min}$ obtained from FEM-based analyses and residuals plots for springs with the spring index *C* equal to: (**a**) 2.5; (**b**) 5; and (**c**) 10.

**Figure 10:**
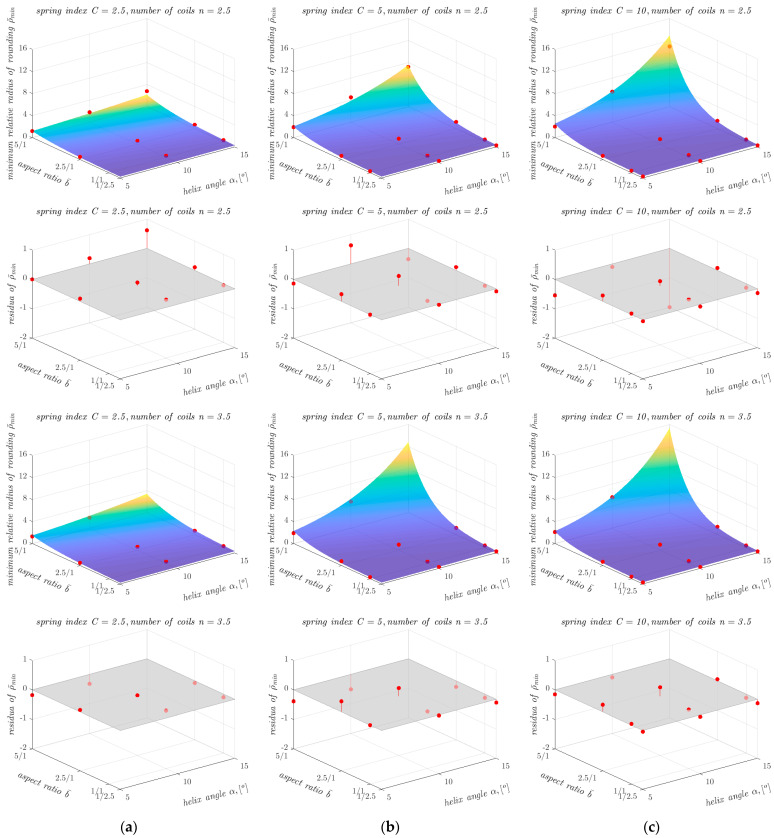
Dependence of ${\bar{\rho }}_{min}$ on the other parameters of the analysed springs (for *n* = 2.5 and *n* = 3.5), together with points showing the values of ${\bar{\rho }}_{min}$ obtained from FEM-based analyses and residuals plots for springs with the spring index *C* equal to: (**a**) 2.5; (**b**) 5; and (**c**) 10.

**Figure 11:**
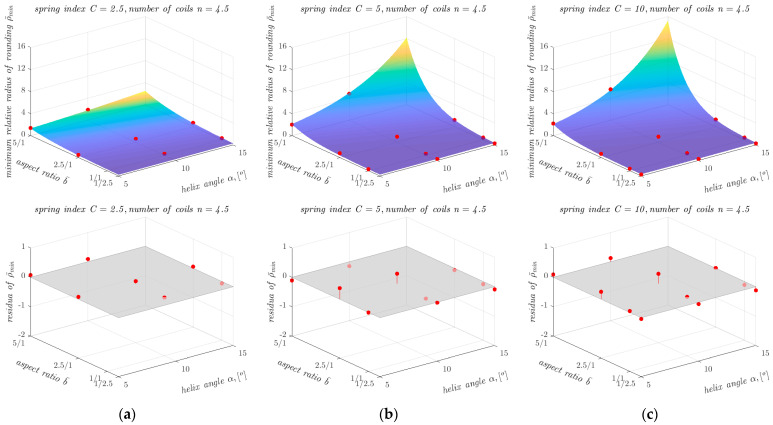
Dependence of ${\bar{\rho }}_{min}$ on the other parameters of the analysed springs (for *n* = 4.5), together with points showing the values of ${\bar{\rho }}_{min}$ obtained from FEM-based analyses and residuals plots for springs with the spring index *C* equal to: (**a**) 2.5; (**b**) 5; and (**c**) 10.

The results obtained with the proposed computational model, described by Equations (2) and (3) and the coefficients shown in Table 7, were compared with the results of the FEM-based analyses and presented graphically in Figure 9, Figure 10 and Figure 11.

Comparing the differences between the ${\bar{\rho }}_{min}$ values obtained from the FEM-based analyses and the ${\bar{\rho }}_{min}$ values obtained with the proposed computational model, it can be seen that the proposed model has a high agreement with the input data, over a wide range of variation in the parameters *C*, $\bar{b}$, *α,* and *n*. It can also be seen that for springs with small values of ${\bar{\rho }}_{min}$, the proposed model generally overestimates the values of this parameter, which is beneficial for the safety of the calculations. The largest differences between the results of the FEM-based analyses and the results of the developed model occur at large values of the helix angle *α* and the aspect ratio $\bar{b}$. However, the relative differences in these cases are small. For example, for a spring with *n* = 1.5, *C* = 10, $\bar{b}\;$= 5/1 and *α* = 15°, the difference in the ${\bar{\rho }}_{min}$ value between the result of the FEM-based analyses and the developed model was 0.8. For a spring with these parameters, the ${\bar{\rho }}_{min}$ value obtained from the FEM-based analyses was 9 (Table 2), so the relative difference did not exceed 9% in this case. The lowest value of the coefficient of determination *R*^2^ was obtained for springs with the spring index *C* = 2.5 and the number of coils *n* = 2.5 and *n* = 3.5. The values of the coefficient of determination *R*^2^ for these springs were 0.92. In all other cases, the values of the coefficient of determination *R*^2^ exceeded 0.95 and in four cases it was 0.99.

**Figure 12:**
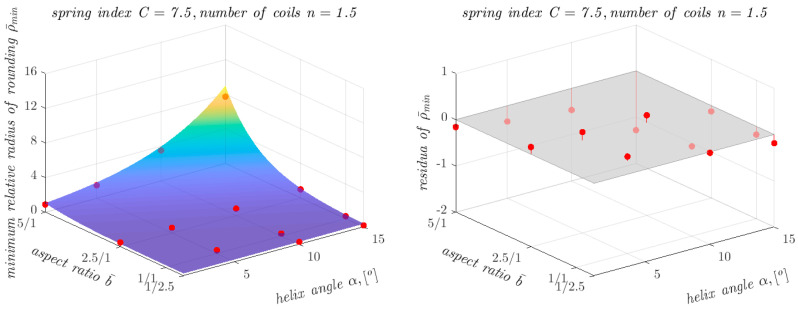
Dependence of ${\bar{\rho }}_{min}$ on the other parameters of the analysed springs made from Equation (2), together with points showing the values of ${\bar{\rho }}_{min}$ obtained from FEM-based analyses and residuals plots for springs with *C* = 7.5 and *n* = 1.5.

In order to validate the developed computational model, a number of additional FEM-based analyses were carried out for springs with the spring index *C* = 7.5 and the number of coils *n* = 1.5. A comparison of the ${\bar{\rho }}_{min}$ values obtained from the FEM-based analyses (red dots) with the course of the function described in Equation (2) is presented in Figure 12.

It can be seen that the results obtained are in good agreement, which is confirmed by the coefficient of determination *R*^2^ value of 0.96.

Table 8 summarises the three extreme values of the absolute differences ${∆}_{abs}$ that were observed between the results of the FEM-based analyses and the results of the proposed computational model. This table also shows the relative differences ${∆}_{rel}$. The values of the radius of roundness of the transition zone obtained from the FEM-based analyses are denoted as ${\bar{\rho }}_{minF}$, and those obtained from the developed approximation model are denoted as ${\bar{\rho }}_{minA}$.

As can be seen, even for the largest observed absolute differences between the results of the FEM-based analyses and the results of the approximation model, the relative differences did not exceed 20% in the worst case.

## 5. Conclusions

This paper presents a new computational model to estimate the geometrical parameters of the transition zones of helical springs with rectangular wire cross-section and closed-end coils, ensuring that the maximum stresses in these zones are reduced to a level corresponding to the stresses in the coils. The model is an approximation and is based on results from more than 350 large deflection FEM-based analyses. The validity of the results of the numerical analyses with respect to stresses was checked using literature data.

The model proposed in this paper, described by Equations (2) and (3) and the data in Table 7, is characterised by a simple formulation and high agreement with the results of FEM-based analyses over a wide range of spring geometric parameters. It is, to the authors’ knowledge, the first such model in the literature to allow the estimation of the minimum radius of the groove rounding in the transition zone of a helical coil spring with a rectangular wire cross-section, for which the stress coefficient factor is 1. This model therefore allows efficient use of the spring material and the space required for its assembly. An additional advantage of the proposed model is that it can be used to calculate springs with wire cross-sectional ratios *b*/*a* both greater and less than one.

The proposed computational model shows good agreement with the results of the FEM-based analyses even in areas with a strongly non-linear relationship between ${\bar{\rho }}_{min}$ values and the other parameters of the analysed springs. In these areas, especially for springs with a small number of coils, in a few cases, large absolute differences were observed between the results, which, however, did not exceed 20% in relation to the values of ${\bar{\rho }}_{min}$ obtained from the FEM-based analyses.

The model can be used for springs with an index between 2.5 and 10, a helix angle between 1° and 15°, and a proportion of the sides of the wire section between 1/2.5 and 5/1. It was developed for the number of coils *n* between 1.5 and 4.5, but in the case of springs with a higher number of coils, the value *n* = 4.5 can be inserted in Equation (3) because, as analyses have shown, at this number of coils, the values of ${\bar{\rho }}_{min}$ stabilise.

Additional analyses carried out during the development of this article, which are not presented here due to their excessive volume, indicated that the stiffness of the analysed springs may differ significantly from the stiffnesses calculated based on the known literature relationships [13,19,21]. This justifies the need to carry out research in this area as well. This research has commenced and will be the subject of a subsequent article.

## Figures and Tables

**Table 1:** Comparison of SCFs values obtained for numerical models with their reference values [27].

Magnitude	$\mathrm{Model}\;\mathrm{with}\;\bar{\rho }=1$	$\mathrm{Model}\;\mathrm{with}\;\bar{\rho }=0.2$
${SCF}_{FEM}$	1.123	1.537
${SCF}_{BFM}$	1.148	1.578
${∆}_{SCF}=\frac{{SCF}_{BFM}-{SCF}_{FEM}}{{SCF}_{BFM}}\times 100\%$	2.2%	2.6%

**Table 2:** Values of ${\bar{\rho }}_{min}$ obtained from FEM-based analyses for *n* = 1.5.

Spring Index *C*	$\mathrm{Aspect}\;\mathrm{Ratio}\;\bar{b}$	$\mathrm{Minimum}\;\mathrm{Relative}\;\mathrm{Radius}\;\mathrm{of}\;\mathrm{Rounding}\;{\bar{\rho }}_{min}$			
		α=1°	α=5°	α=10°	α=15°
2.5	5/1	–	1.0	1.4	1.9
	2.5/1	–	0.4	0.5	0.6
	1/1	–	–	0.2	0.2
	1/2.5	–	–	–	–
5	5/1	0.9	1.4	3.1	5.3
	2.5/1	–	0.7	0.8	1.0
	1/1	–	0.3	0.3	0.3
	1/2.5	–	–	0.2	0.2
10	5/1	0.9	1.5	3.7	9
	2.5/1	0.5	0.6	0.8	1.0
	1/1	–	0.4	0.3	0.3
	1/2.5	–	0.3	0.3	0.2

**Table 3:** Values of ${\bar{\rho }}_{min}$ obtained from FEM-based analyses for *n* = 2.5.

Spring Index *C*	$\mathrm{Aspect}\;\mathrm{Ratio}\;\bar{b}$	$\mathrm{Minimum}\;\mathrm{Relative}\;\mathrm{Radius}\;\mathrm{of}\;\mathrm{Rounding}\;{\bar{\rho }}_{min}$			
		α=1°	α=5°	α=10°	α=15°
2.5	5/1	–	1.2	1.8	2.8
	2.5/1	–	0.5	0.6	0.7
	1/1	–	–	0.3	0.3
	1/2.5	–	–	–	–
5	5/1	1.0	1.9	4.5	7.2
	2.5/1	–	0.7	1.0	1.2
	1/1	–	0.3	0.3	0.4
	1/2.5	–	–	0.3	0.3
10	5/1	1.0	2.0	5.5	10.9
	2.5/1	0.6	0.7	0.9	1.4
	1/1	–	0.4	0.4	0.4
	1/2.5	–	0.3	0.3	0.3

**Table 4:** Values of ${\bar{\rho }}_{min}$ obtained from FEM-based analyses for *n* = 3.5.

Spring Index *C*	$\mathrm{Aspect}\;\mathrm{Ratio}\;\bar{b}$	$\mathrm{Minimum}\;\mathrm{Relative}\;\mathrm{Radius}\;\mathrm{of}\;\mathrm{Rounding}\;{\bar{\rho }}_{min}$			
		α=1°	α=5°	α=10°	α=15°
2.5	5/1	–	1.3	1.9	buckling
	2.5/1	–	0.5	0.6	0.7
	1/1	–	–	0.3	0.3
	1/2.5	–	–	–	–
5	5/1	1.0	1.9	4.8	buckling
	2.5/1	–	0.8	1.0	1.2
	1/1	–	0.3	0.3	0.4
	1/2.5	–	–	0.3	0.3
10	5/1	1.0	2.1	5.6	buckling
	2.5/1	0.6	0.7	1.0	1.4
	1/1	–	0.4	0.4	0.4
	1/2.5	–	0.3	0.3	0.3

**Table 5:** Values of ${\bar{\rho }}_{min}$ obtained from FEM-based analyses for *n* = 4.5.

Spring Index *C*	$\mathrm{Aspect}\;\mathrm{Ratio}\;\bar{b}$	$\mathrm{Minimum}\;\mathrm{Relative}\;\mathrm{Radius}\;\mathrm{of}\;\mathrm{Rounding}\;{\bar{\rho }}_{min}$			
		α=1°	α=5°	α=10°	α=15°
2.5	5/1	–	1.4	1.9	buckling
	2.5/1	–	0.5	0.6	0.7
	1/1	–	–	0.3	0.3
	1/2.5	–	–	–	–
5	5/1	1.0	2.0	4.8	buckling
	2.5/1	–	0.8	1.0	1.2
	1/1	–	0.3	0.3	0.4
	1/2.5	–	–	0.3	0.3
10	5/1	1.0	2.2	5.6	buckling
	2.5/1	0.6	0.7	1.0	1.3
	1/1	–	0.4	0.4	0.4
	1/2.5	–	0.3	0.3	0.3

**Table 6:** Values of the coefficients *c_i_*.

Spring Index *C*	Number of Coils *n*	*c*_1_ × 10^3^	*c*_2_ × 10^3^	*c*_3_ × 10^3^	*c*_4_ × 10^3^	*R* ^2^	MSE
2.5	1.5	21.61	68.85	660.8	233.2	0.9886	0.0256
	2.5	21.49	87.23	690.8	225.3	0.9820	0.0484
	3.5	26.49	78.44	670.6	209.3	0.9739	0.0491
	4.5	28.06	75.51	668.6	207.7	0.9770	0.0472
5	1.5	10.92	138.3	817.3	232.4	0.9618	0.0768
	2.5	14.71	130.7	844.5	248.8	0.9854	0.2572
	3.5	5.219	186.9	975.4	259.2	0.9406	0.1071
	4.5	5.035	186.4	985.5	264.0	0.9425	0.1062
10	1.5	3.187	190.6	1010	322.3	0.9978	0.1139
	2.5	5.629	166.1	1012	323.3	0.9934	0.2407
	3.5	2.817	214.2	1079	328.1	0.9894	0.1493
	4.5	2.724	209.0	1095	330.7	0.9452	0.1010

**Table 7:** Values of the determined constants of Equation (3).

*c_i_*	*p*_00_ × 10^3^	*p*_10_ × 10^3^	*p*_01_ × 10^3^	*p*_20_ × 10^3^	*p*_11_ × 10^3^	*p*_02_ × 10^3^	*p*_21_ × 10^3^	*p*_12_ × 10^3^	*p*_03_ × 10^3^	*R* ^2^	MSE
*c*_1_	−10.61	1.603	40.84	−0.3275	−3.916	−10.76	0.3358	−0.1104	1.223	0.9739	0.0030
*c*_2_	245.6	17.98	−288.3	0.2936	9.604	99.83	−1.269	1.247	−11.85	0.9746	0.0168
*c*_3_	901.8	−0.9397	−421.3	4.021	47.64	124.2	−4.208	1.536	−15.12	0.9931	0.0270
*c*_4_	304.1	−41.49	−20.83	4.046	17.56	−8.525	−1.255	−0.040	0.9389	0.9974	0.0047

**Table 8:** Comparison of extreme differences between the results of FEM-based analyses and the proposed model.

$\bar{b}$	*C*	*α* [°]	*n*	${\bar{\rho }}_{minF}$	${\bar{\rho }}_{minA}$	${∆}_{abs}$	${∆}_{rel}$ [%]
5	10	15	2.5	10.9	12.9	−2	−18
5	7.5	15	1.5	7.7	9	−1.3	−17
5	10	15	1.5	9	8.2	0.8	9

## Data Availability

The raw data supporting the conclusions of this article will be made available by the authors on request.
